# (2,2′-Bi­pyridine-κ^2^ *N*,*N*′)di­chloridopalladium(II) 1,4-dioxane hemisolvate

**DOI:** 10.1107/S1600536814009507

**Published:** 2014-05-17

**Authors:** Ricardo Alfredo Gutiérrez Márquez, Carmela Crisóstomo-Lucas, David Morales-Morales, Simón Hernández-Ortega

**Affiliations:** a Instituto de Química, Universidad Nacional Autónoma de México, Circuito Exterior, Ciudad Universitaria, Coyoacán, c.p. 04510, México, DF, Mexico

## Abstract

The asymmetric unit of the title compound, [PdCl_2_(C_10_H_8_N_2_)]·0.5C_4_H_8_O_2_, consists of one Pd^II^ complex mol­ecule and a half-mol­ecule of 1,4-dioxane, the complete mol­ecule being generated by inversion symmetry. The Pd^II^ atom has an almost square-planar coordination formed by the 2,2′-bi­pyridine ligand and two chloride ligands. Two intra­molecular C—H⋯Cl hydrogen bonds occur. In the crystal, the Pd^II^ complex and 1,4-dioxane mol­ecules are connected by C—H⋯O hydrogen bonds, forming a layer parallel to (10-1). Within the layer, weak π–π inter­actions [centroid–centroid distance = 3.817 (4) Å] are observed between the pyridine rings.

## Related literature   

For related structures, see: Maekawa *et al.* (1991 ▶); Vicente *et al.* (1997 ▶); Kim *et al.* (2009 ▶). For palladium complexes with chelate ligands, see: Pointillart *et al.* (2007 ▶); Pazderski *et al.* (2006 ▶); Ferbinteanu *et al.* (1998 ▶). For puckering parameters, see: Cremer & Pople (1975 ▶). 
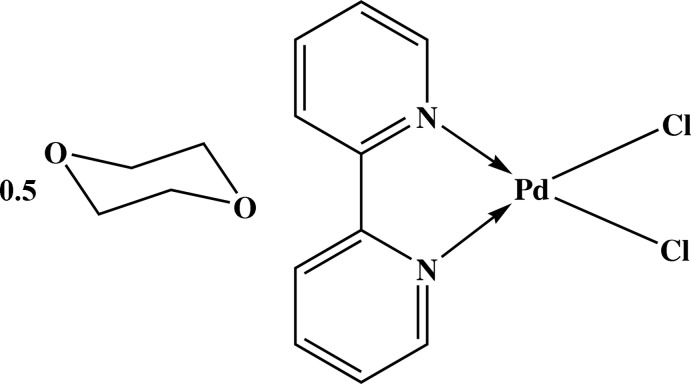


## Experimental   

### 

#### Crystal data  

[PdCl(C_10_H_8_N_2_)_2_]·0.5C_4_H_8_O_2_
*M* *_r_* = 377.54
Monoclinic, 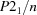

*a* = 7.2416 (5) Å
*b* = 14.6215 (10) Å
*c* = 12.9562 (9) Å
β = 105.423 (2)°
*V* = 1322.44 (16) Å^3^
*Z* = 4
Mo *K*α radiation
μ = 1.80 mm^−1^
*T* = 298 K
0.40 × 0.07 × 0.06 mm

#### Data collection  

Bruker APEXII CCD area-detector diffractometer
Absorption correction: multi-scan (*SADABS*; Bruker, 2007 ▶) *T*_min_ = 0.365, *T*_max_ = 0.906
7244 measured reflections
2414 independent reflections
1813 reflections with *I* > 2σ(*I*)
*R*_int_ = 0.055

#### Refinement  

*R*[*F* ^2^ > 2σ(*F* ^2^)] = 0.051
*wR*(*F* ^2^) = 0.153
*S* = 1.03
2414 reflections
163 parameters
H-atom parameters constrained
Δρ_max_ = 1.49 e Å^−3^
Δρ_min_ = −1.13 e Å^−3^

### 

Data collection: *APEX2* (Bruker, 2007 ▶); cell refinement: *SAINT* (Bruker, 2007 ▶); data reduction: *SAINT*; program(s) used to solve structure: *SHELXTL* (Sheldrick, 2008 ▶); program(s) used to refine structure: *SHELXL2013* (Sheldrick, 2008 ▶); molecular graphics: *ORTEP-3* (Farrugia, 2012 ▶) and *SHELXTL*; software used to prepare material for publication: *SHELXTL* and *PLATON* (Spek, 2009 ▶).

## Supplementary Material

Crystal structure: contains datablock(s) I, New_Global_publ_block. DOI: 10.1107/S1600536814009507/is5358sup1.cif

Structure factors: contains datablock(s) I. DOI: 10.1107/S1600536814009507/is5358Isup2.hkl

CCDC reference: 999627

Additional supporting information:  crystallographic information; 3D view; checkCIF report

## Figures and Tables

**Table 1:** Hydrogen-bond geometry (Å, °)

*D*—H⋯*A*	*D*—H	H⋯*A*	*D*⋯*A*	*D*—H⋯*A*
C3—H3⋯O1	0.93	2.55	3.471 (9)	170
C5—H5⋯O1^i^	0.93	2.57	3.464 (8)	163
C9—H9⋯O1	0.93	2.66	3.587 (8)	174
C6—H6⋯Cl2	0.93	2.65	3.239 (7)	122
C12—H12⋯Cl1	0.93	2.65	3.238 (7)	122

Symmetry code: (i) 
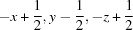
.

**Fig. 1.**
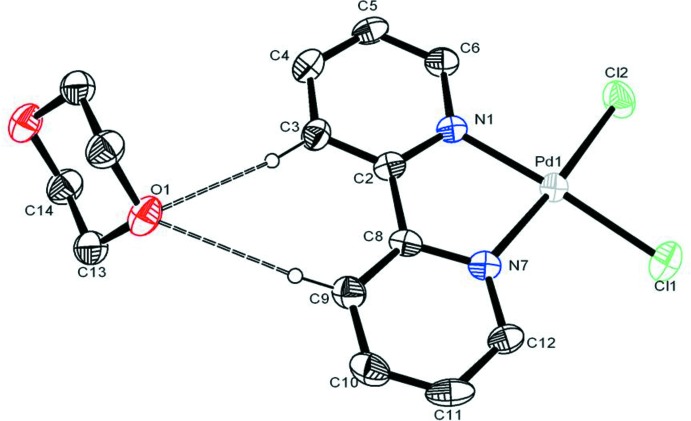
The structure of the title compound, the displacement ellipsoids are drawn at the 40% of probability. Only the hydrogen atom involved in intermolecular interaction are drawn.

**Fig. 2.**
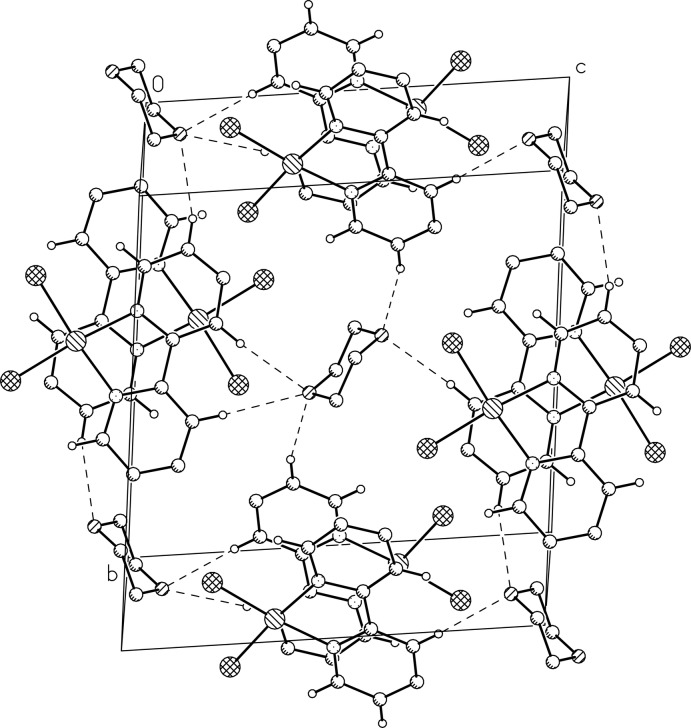
Crystal packing diagram of the title compound. Hydrogen bonds are drawn as dashed lines.

## supplementary crystallographic information

### 1. Comment

In recent years the use of chelate ligands of the type N—N has been of great interest due to the versatility of its applications. This is particularly true in the case of supramolecular chemistry and crystal engineering, where these kind of chelates are often used as blocking ligands and the study of their metallic derivatives in the solid state has revealed the tremendous importance of non-covalent interactions and advanced the understanding of the reactivity of these species (Pointillart *et al.*, 2007; Pazderski *et al.*, 2006). Complexes of group 10 elements with these ligands often present square planar geometries, and in some cases, they can exhibit interesting dimeric and trimeric structures (Ferbinteanu *et al.* 1998). The coordination complex [PdCl_2_(C_10_H_8_N_2_)] has been described before (Maekawa *et al.*, 1991), and as CH_2_Cl_2_ solvate (Vicente *et al.*, 1997; Kim *et al.*, 2009). Here, we describe the structure of the title complex and its interaction with 1,4-dioxane as solvate.

The Pd complex crystallizes as 1,4-dioxane solvate and the solvent is determined as a half-molecule in the asymmetric unit; the symmetry operation 1 - *x*, 1 - *y*, 1 - *z* is necessary to generate the whole molecule. The title compound has similar values of bond distances and angles to the previously described CH_2_Cl_2_ solvate (Maekawa *et al.*, 1991; Vicente *et al.*, 1997; Kim *et al.*, 2009). According to the Cremer & Pople puckering parameters (Cremer & Pople, 1975), the dioxane molecule has a chair conformation [Q=0.553 (8), θ=180.00 (1)°, φ =0°]. In the asymmetric unit, the O atom is bonded to H3—C3 and H9—C9 in a bifurcated fashion (Fig. 1). When the symmetry code (1 - *x*, 1 - *y*, 1 - *z*) is applied, a centrosymmetric structure of Pd complex–1,4-dioxane (2/1) is generated. In addition, the O atom is linked by O···H5—C5(bipy) (Table 1 and Fig. 2). As a result of these interactions, the bipyridine complexes are bonded by a weak π–π stacking interaction [*Cg*1(N1/C2–C6)···*Cg*2(N7/C8–C12) 3.817 (4) Å].

### 2. Experimental

To a solution of [Pd(MeCN)_2_Cl_2_] (0.13 g, 0.638 mmol) in acetone (10 ml), 2,2'-bipyridine (0.1 g, 0.64 mmol) was added under stirring. The resulting orange solution was allowed to react for 2 h under stirring at room temperature. After this time the solution was filtered and the solvent taken off under vacuum to produce a yellow solid of [(bipy)PdCl_2_]. Crystals suitable for X-ray diffraction experiments were obtained from a dimethylformamide/dioxane solvent system at room temperature. ^1^H NMR (DMSO-d_6_): δ 7.83 (t, 1H, CH), 8.38 (t, 1H, CH), 8.60 (d, 1H, CH), 9.14 (d, 1H, CH); ^13^C{^1^H}NMR (DMSO-D_6_): δ 124.3 (s, CH), 127.8 (s, CH), 141.7 (s, CH). 150.1 (s, CH). 156.9 (s, C).

### 3. Refinement

H atoms were included in calculated positions (C—H = 0.97 Å for methylene and C—H = 0.96 Å for aromatic ring), and refined using a riding model with *U*_iso_(H) = 1.2*U*_eq_(C). 4 reflections were omitted from the final refinement.

### Crystal data

**Table:**

[PdCl(C_10_H_8_N_2_)_2_]·0.5C_4_H_8_O_2_	*F*(000) = 744
*M**_r_* = 377.54	*D*_x_ = 1.896 Mg m^−^^3^
Monoclinic, *P*2_1_/*n*	Mo *K*α radiation, λ = 0.71073 Å
*a* = 7.2416 (5) Å	Cell parameters from 4271 reflections
*b* = 14.6215 (10) Å	θ = 2.8–25.4°
*c* = 12.9562 (9) Å	µ = 1.80 mm^−^^1^
β = 105.423 (2)°	*T* = 298 K
*V* = 1322.44 (16) Å^3^	Prism, yellow
*Z* = 4	0.40 × 0.07 × 0.06 mm

### Data collection

**Table:**

Bruker APEXII CCD area-detector diffractometer	1813 reflections with *I* > 2σ(*I*)
Detector resolution: 0.83 pixels mm^-1^	*R*_int_ = 0.055
ω scans	θ_max_ = 25.4°, θ_min_ = 2.8°
Absorption correction: multi-scan (*SADABS*; Bruker, 2007)	*h* = −8→8
*T*_min_ = 0.365, *T*_max_ = 0.906	*k* = −17→16
7244 measured reflections	*l* = −14→15
2414 independent reflections	

### Refinement

**Table:**

Refinement on *F*^2^	0 restraints
Least-squares matrix: full	Hydrogen site location: inferred from neighbouring sites
*R*[*F*^2^ > 2σ(*F*^2^)] = 0.051	H-atom parameters constrained
*wR*(*F*^2^) = 0.153	*w* = 1/[σ^2^(*F*_o_^2^) + (0.0933*P*)^2^] where *P* = (*F*_o_^2^ + 2*F*_c_^2^)/3
*S* = 1.03	(Δ/σ)_max_ < 0.001
2414 reflections	Δρ_max_ = 1.49 e Å^−^^3^
163 parameters	Δρ_min_ = −1.13 e Å^−^^3^

### Special details

**Table:**

Geometry. All e.s.d.'s (except the e.s.d. in the dihedral angle between two l.s. planes) are estimated using the full covariance matrix. The cell e.s.d.'s are taken into account individually in the estimation of e.s.d.'s in distances, angles and torsion angles; correlations between e.s.d.'s in cell parameters are only used when they are defined by crystal symmetry. An approximate (isotropic) treatment of cell e.s.d.'s is used for estimating e.s.d.'s involving l.s. planes.

### Fractional atomic coordinates and isotropic or equivalent isotropic displacement parameters (Å^2^)

**Table:**

	*x*	*y*	*z*	*U*_iso_*/*U*_eq_	
Pd1	0.16709 (6)	0.48172 (3)	−0.14236 (4)	0.0377 (2)	
Cl1	0.1430 (3)	0.56553 (14)	−0.29371 (14)	0.0636 (5)	
Cl2	0.0446 (3)	0.35687 (14)	−0.24462 (15)	0.0657 (5)	
N1	0.1942 (6)	0.4175 (3)	−0.0003 (4)	0.0376 (12)	
C2	0.2757 (9)	0.4690 (4)	0.0871 (5)	0.0387 (15)	
C3	0.3108 (10)	0.4325 (5)	0.1885 (5)	0.0487 (16)	
H3	0.3678	0.4679	0.2481	0.058*	
C4	0.2607 (10)	0.3433 (5)	0.2008 (6)	0.0542 (18)	
H4	0.2840	0.3179	0.2688	0.065*	
C5	0.1779 (9)	0.2927 (5)	0.1140 (5)	0.0512 (17)	
H5	0.1436	0.2324	0.1221	0.061*	
C6	0.1439 (9)	0.3305 (5)	0.0125 (6)	0.0496 (16)	
H6	0.0858	0.2955	−0.0472	0.060*	
N7	0.2849 (7)	0.5840 (3)	−0.0419 (4)	0.0401 (12)	
C8	0.3249 (8)	0.5628 (4)	0.0654 (5)	0.0382 (14)	
C9	0.4075 (9)	0.6253 (5)	0.1418 (5)	0.0493 (16)	
H9	0.4329	0.6098	0.2138	0.059*	
C10	0.4534 (10)	0.7111 (5)	0.1131 (6)	0.0587 (19)	
H10	0.5092	0.7541	0.1650	0.070*	
C11	0.4154 (10)	0.7319 (5)	0.0072 (7)	0.059 (2)	
H11	0.4461	0.7894	−0.0139	0.071*	
C12	0.3315 (9)	0.6679 (4)	−0.0688 (6)	0.0468 (16)	
H12	0.3064	0.6833	−0.1408	0.056*	
O1	0.4638 (7)	0.5658 (4)	0.4170 (4)	0.0603 (13)	
C13	0.6526 (11)	0.5477 (6)	0.4826 (6)	0.0583 (19)	
H13A	0.6822	0.5899	0.5426	0.070*	
H13B	0.7444	0.5577	0.4413	0.070*	
C14	0.6707 (11)	0.4527 (6)	0.5235 (6)	0.062 (2)	
H14A	0.6504	0.4105	0.4637	0.075*	
H14B	0.7994	0.4432	0.5688	0.075*	

### Atomic displacement parameters (Å^2^)

**Table:**

	*U*^11^	*U*^22^	*U*^33^	*U*^12^	*U*^13^	*U*^23^
Pd1	0.0384 (3)	0.0347 (4)	0.0405 (4)	0.00612 (19)	0.0114 (2)	−0.0008 (2)
Cl1	0.0829 (13)	0.0635 (13)	0.0483 (10)	0.0124 (10)	0.0241 (9)	0.0100 (9)
Cl2	0.0785 (12)	0.0538 (12)	0.0561 (11)	−0.0033 (9)	0.0025 (9)	−0.0138 (9)
N1	0.034 (2)	0.035 (3)	0.047 (3)	0.004 (2)	0.016 (2)	0.000 (2)
C2	0.038 (3)	0.036 (4)	0.048 (4)	0.006 (3)	0.023 (3)	−0.003 (3)
C3	0.053 (4)	0.054 (5)	0.041 (4)	0.000 (3)	0.017 (3)	0.002 (3)
C4	0.062 (4)	0.056 (5)	0.050 (4)	0.006 (3)	0.024 (3)	0.008 (4)
C5	0.058 (4)	0.034 (4)	0.069 (5)	0.000 (3)	0.029 (4)	0.009 (4)
C6	0.048 (4)	0.041 (4)	0.061 (4)	0.001 (3)	0.018 (3)	0.000 (3)
N7	0.036 (3)	0.037 (3)	0.051 (3)	0.004 (2)	0.016 (2)	−0.002 (3)
C8	0.034 (3)	0.033 (3)	0.051 (4)	0.000 (3)	0.019 (3)	−0.009 (3)
C9	0.054 (4)	0.046 (4)	0.051 (4)	−0.002 (3)	0.020 (3)	−0.010 (3)
C10	0.065 (4)	0.044 (4)	0.070 (5)	−0.008 (3)	0.023 (4)	−0.025 (4)
C11	0.055 (4)	0.025 (3)	0.108 (6)	0.002 (3)	0.043 (4)	0.000 (4)
C12	0.051 (4)	0.034 (4)	0.060 (4)	0.003 (3)	0.022 (3)	0.005 (3)
O1	0.078 (3)	0.055 (3)	0.052 (3)	0.013 (3)	0.023 (3)	0.013 (3)
C13	0.066 (5)	0.058 (5)	0.055 (4)	−0.007 (4)	0.022 (4)	−0.006 (4)
C14	0.062 (5)	0.062 (5)	0.064 (5)	0.008 (4)	0.018 (4)	0.014 (4)

### Geometric parameters (Å, º)

**Table:**

Pd1—N7	2.017 (5)	C8—C9	1.363 (9)
Pd1—N1	2.029 (5)	C9—C10	1.374 (10)
Pd1—Cl1	2.2793 (18)	C9—H9	0.9300
Pd1—Cl2	2.2912 (19)	C10—C11	1.360 (11)
N1—C6	1.345 (8)	C10—H10	0.9300
N1—C2	1.357 (8)	C11—C12	1.376 (9)
C2—C3	1.377 (9)	C11—H11	0.9300
C2—C8	1.463 (9)	C12—H12	0.9300
C3—C4	1.375 (10)	O1—C14^i^	1.420 (8)
C3—H3	0.9300	O1—C13	1.430 (9)
C4—C5	1.346 (10)	C13—C14	1.479 (11)
C4—H4	0.9300	C13—H13A	0.9700
C5—C6	1.387 (9)	C13—H13B	0.9700
C5—H5	0.9300	C14—O1^i^	1.420 (8)
C6—H6	0.9300	C14—H14A	0.9700
N7—C12	1.344 (8)	C14—H14B	0.9700
N7—C8	1.377 (8)		
			
N7—Pd1—N1	80.5 (2)	C9—C8—C2	124.8 (6)
N7—Pd1—Cl1	94.55 (16)	N7—C8—C2	114.1 (5)
N1—Pd1—Cl1	174.96 (15)	C8—C9—C10	120.4 (7)
N7—Pd1—Cl2	175.00 (15)	C8—C9—H9	119.8
N1—Pd1—Cl2	94.89 (15)	C10—C9—H9	119.8
Cl1—Pd1—Cl2	90.09 (7)	C11—C10—C9	118.5 (7)
C6—N1—C2	119.6 (5)	C11—C10—H10	120.7
C6—N1—Pd1	125.8 (5)	C9—C10—H10	120.7
C2—N1—Pd1	114.6 (4)	C10—C11—C12	120.2 (7)
N1—C2—C3	120.6 (6)	C10—C11—H11	119.9
N1—C2—C8	115.7 (5)	C12—C11—H11	119.9
C3—C2—C8	123.7 (6)	N7—C12—C11	121.9 (6)
C4—C3—C2	119.4 (6)	N7—C12—H12	119.0
C4—C3—H3	120.3	C11—C12—H12	119.0
C2—C3—H3	120.3	C14^i^—O1—C13	109.1 (6)
C5—C4—C3	119.8 (7)	O1—C13—C14	111.5 (6)
C5—C4—H4	120.1	O1—C13—H13A	109.3
C3—C4—H4	120.1	C14—C13—H13A	109.3
C4—C5—C6	120.0 (6)	O1—C13—H13B	109.3
C4—C5—H5	120.0	C14—C13—H13B	109.3
C6—C5—H5	120.0	H13A—C13—H13B	108.0
N1—C6—C5	120.6 (6)	O1^i^—C14—C13	111.4 (6)
N1—C6—H6	119.7	O1^i^—C14—H14A	109.3
C5—C6—H6	119.7	C13—C14—H14A	109.3
C12—N7—C8	117.8 (5)	O1^i^—C14—H14B	109.3
C12—N7—Pd1	127.0 (5)	C13—C14—H14B	109.3
C8—N7—Pd1	115.1 (4)	H14A—C14—H14B	108.0
C9—C8—N7	121.1 (6)		

Symmetry code: (i) −*x*+1, −*y*+1, −*z*+1.

### Hydrogen-bond geometry (Å, º)

**Table:**

*D*—H···*A*	*D*—H	H···*A*	*D*···*A*	*D*—H···*A*
C3—H3···O1	0.93	2.55	3.471 (9)	170
C5—H5···O1^ii^	0.93	2.57	3.464 (8)	163
C9—H9···O1	0.93	2.66	3.587 (8)	174
C6—H6···Cl2	0.93	2.65	3.239 (7)	122
C12—H12···Cl1	0.93	2.65	3.238 (7)	122

Symmetry code: (ii) −*x*+1/2, *y*−1/2, −*z*+1/2.
